# Nucleoside-Modified mRNA Vaccines Protect IFNAR^–/–^ Mice against Crimean-Congo Hemorrhagic Fever Virus Infection

**DOI:** 10.1128/jvi.01568-21

**Published:** 2022-02-09

**Authors:** Sofia Appelberg, Lijo John, Norbert Pardi, Ákos Végvári, Sándor Bereczky, Gustaf Ahlén, Vanessa Monteil, Samir Abdurahman, Flora Mikaeloff, Mitchell Beattie, Ying Tam, Matti Sällberg, Ujjwal Neogi, Drew Weissman, Ali Mirazimi

**Affiliations:** a Public Health Agency of Sweden, Solna, Sweden; b National Veterinary Institute, Uppsala, Sweden; c Department of Medicine, Perelman School of Medicine, University of Pennsylvania, Philadelphia, Pennsylvania, USA; d Division of Chemistry I, Department of Medical Biochemistry and Biophysics, Karolinska Institute t, Stockholm, Sweden; e Division of Clinical Microbiology, Department of Laboratory Medicine, Karolinska Institute t, Stockholm, Sweden; f Acuitas Therapeutics, Vancouver, British Columbia, Canada; University of Kentucky College of Medicine

**Keywords:** Crimean-Congo hemorrhagic fever virus, Gn, Gc, N, IFNAR mice, T-cell immunity, mRNA vaccine, neutralizing antibodies

## Abstract

Crimean-Congo hemorrhagic fever (CCHF), caused by Crimean-Congo hemorrhagic fever virus (CCHFV), is on the World Health Organizations’ list of prioritized diseases and pathogens. With global distribution, high fatality rate, and no approved vaccine or effective treatment, CCHF constitutes a threat against global health. In the current study, we demonstrate that vaccination with nucleoside-modified mRNA-lipid nanoparticles (mRNA-LNP), encoding for the CCHFV nucleoprotein (N) or glycoproteins (GcGn) protect IFNAR^−/−^ mice against lethal CCHFV infection. In addition, we found that both mRNA-LNP induced strong humoral and cellular immune responses in IFNAR^−/−^ and immunocompetent mice and that neutralizing antibodies are not necessary for protection. When evaluating immune responses induced by immunization including CCHFV Gc and Gn antigens, we found the Gc protein to be more immunogenic compared with the Gn protein. Hepatic injury is prevalent in CCHF and contributes to the severity and mortality of the disease in humans. Thus, to understand the immune response in the liver after infection and the potential effect of the vaccine, we performed a proteomic analysis on liver samples from vaccinated and control mice after CCHFV infection. Similar to observations in humans, vaccination affected the metabolic pathways. In conclusion, this study shows that a CCHFV mRNA-LNP vaccine, based on viral nucleo- or glycoproteins, mediate protection against CCHFV induced disease. Consequently, genetic immunization is an attractive approach to prevent disease caused by CCHFV and we believe we have necessary evidence to bring this vaccine platform to the next step in the development of a vaccine against CCHFV infection.

**IMPORTANCE** Crimean-Congo hemorrhagic fever virus (CCHFV) is a zoonotic pathogen causing Crimean-Congo hemorrhagic fever (CCHF), a severe fever disease. CCHFV has a wide distribution and is endemic in several areas around the world. Cases of CCHF are also being reported in new areas, indicating an expansion of the disease, which is of high concern. Dispersion of the disease, high fatality rate, and no approved vaccine makes CCHF a threat to global health. The development of a vaccine is thus of great importance. Here we show 100% protection against lethal CCHFV infection in mice immunized with mRNA-LNP encoding for different CCHFV proteins. The vaccination showed both robust humoral and cellular immunity. mRNA-LNP vaccines combine the ability to induce an effective immune response, the safety of a transient carrier, and the flexibility of genetic vaccines. This and our results from the current study support the development of a mRNA-LNP based vaccine against CCHFV.

## INTRODUCTION

Crimean-Congo hemorrhagic fever orthonairovirus (CCHFV), a single-stranded, negative sense RNA virus belonging to the family *Nairoviridae*, is the causative agent of Crimean-Congo hemorrhagic fever (CCHF). Occurrence of the virus coincides with the distribution of its primary vector and reservoir, *Hyalomma* ticks. Thus, CCHF is geographically widespread and present in Asia, Africa, the Middle East and Southeast Europe (1). Recently, Spain has reported cases of CCHF (2) and in 2018, *Hyalomma* ticks were found as far north as Sweden (3). Global climate changes may be a cause for the introduction of the *Hyalomma* tick to new areas and consequently, spreading of the disease.

Mode of transmission of the virus and hence the disease is tick bites; handling of infected livestock and/or care of CCHFV infected patients. In humans, CCHFV causes a febrile-illness with a wide span of symptoms and severity of the disease. Some cases are mild with fever and muscle pain, while in others the infection causes severe disease with hemorrhagic manifestations. The global fatality rate for CCHF is approximately 32.2% (4) and currently there are no effective treatments or approved vaccines against CCHF. The extensive spread of the virus, mode of transmission, and severity of the disease make CCHF a significant threat to global health.

In recent years, nucleoside-modified mRNA have emerged as a promising therapeutic modality due to significant advances in the engineering of mRNA sequences, progress of rapid and large-scale cGMP production, and development of efficient and safe mRNA vaccine delivery materials (5), with ionizable lipid-containing nanoparticles (LNP) being the most widely used (6). Multiple vaccine studies have successfully used the nucleoside-modified mRNA-LNP platform against numerous infectious pathogens (7–16), and recently the advantage of this vaccine concept has been demonstrated during the development of a vaccine against SARS-CoV-2 (17–25). Compared with other vaccination methods, nucleoside-modified mRNA-LNP has several advantages, including that it is nonintegrating, noninfectious, it can express protein with high efficiency, and small doses are efficient to induce a robust, protective immune response. Moreover, this delivery system, and the nucleoside modification of the RNA, increases the intracellular stability and dampens innate immune reactions.

Even though nucleoside-modified mRNA-LNP-based vaccine candidates have been used in vaccine studies against several infectious diseases (7–16), it has not been evaluated against CCHFV infection. However, naked mRNA, based on the S-segment of CCHFV (Ank-2 strain), has previously been used to immunize IFNAR^−/−^ mice prior to CCHFV challenge (26), with a difference in success based on the immunization schedule.

In the current study, we assessed two different CCHFV immunogens in nucleoside-modified mRNA-LNP vaccines in both immunocompromised and immunocompetent mice. We demonstrated protective efficacy and characterized the humoral and cellular immune responses induced by our vaccine candidates. In addition, we conducted proteomic analysis on liver samples from control and vaccinated CCHFV-infected mice. To our knowledge, this is the first time a nucleoside-modified mRNA-LNP vaccine has been evaluated for protection against CCHFV infection and where the effect of the vaccination has been measured by large-scale protein analyses.

## RESULTS

### Design of nucleoside modified RNA vaccine against CCHFV.

We have previously shown that our experimental DNA vaccine platform expressing either the glycoproteins or nucleoprotein genes of CCHFV elicits protective immunity against CCHFV infection when given in combination (27). However, to further explore the best and most efficient CCHFV vaccine, based on both antigen and vaccine platform, we designed and generated two nucleoside-modified mRNA-LNP vaccines encoding different antigens, either CCHFV IbAr10200 glycoproteins (Gc and Gn) or nucleoprotein (N). The modified mRNA was synthesized enzymatically with 1-methylpseudouridine (m1Ψ), an enzymatic cap1, and a 101-nucleotide long poly(A) tail and packaged into lipid nanoparticles for vaccination. Transfection of HEK 293T cells with the two modified mRNA-LNP resulted in efficient protein expression as analyzed using Western blot (data not shown).

### Nucleoside-modified mRNA-LNP vaccinated IFNAR^–/–^ mice survive CCHFV infection.

**FIG 1:**
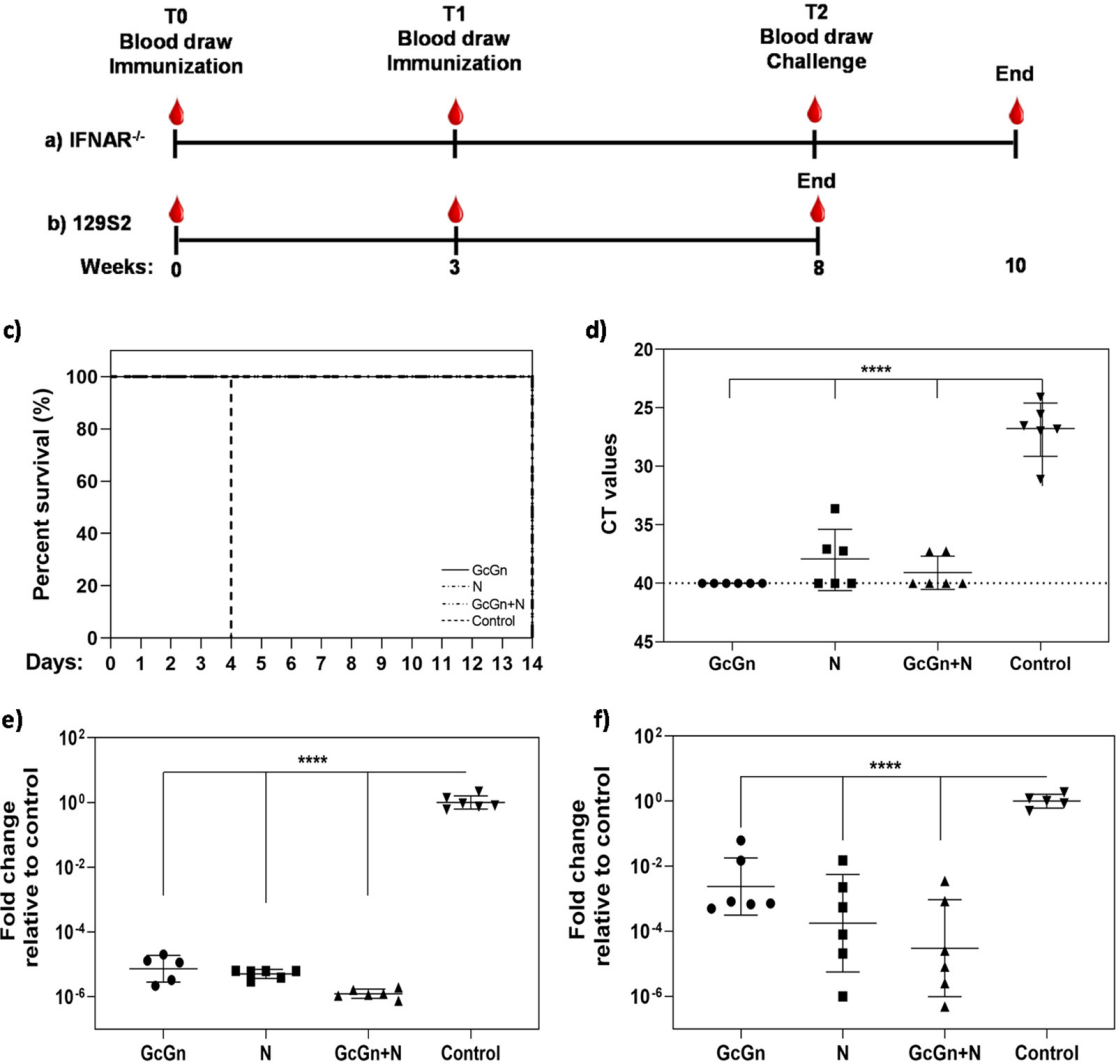
Survival and viral RNA in mRNA-LNP CCHFV-infected IFNAR^−/−^ mice. Schematic drawing illustrating the immunization schedule for (a) A129 IFNAR^−/−^ mice and (b) immunocompetent 129S2 mice. Each mouse received two immunizations with 3 weeks between. The mice received 10 μg of each specified mRNA-LNP administered through intradermal injections. Five weeks post last immunization, all immunocompetent mice were euthanized, while the IFNAR^−/−^ mice were challenged with CCHFV IbAr10200 via intraperitoneal injection and followed for 2 weeks. (c) Kaphlan-Meire graph showing survival of vaccinated and control IFNAR^−/−^ mice after CCHFV challenge (GcGn, N, GcGn+N, and control). At the time of euthanasia, viral RNA in (d) serum, (e) spleen, and (f) liver was measured using qRT-PCR. For serum, data is shown as mean CT values ± geometric standard deviation. Dashed line indicate limit of detection. For spleen and liver, the graphs display fold change ± geometric standard deviation relative to the control group. ****P < 0.0001. *P values* calculated with one-way ANOVA with Dunnett’s multiple comparison test.

Twenty-four female A129 IFNAR^−/−^ mice, divided into four groups (*n* = 6), received immunizations at two different time points (Fig. 1a). Each mouse was given intradermal injections with 10 μg of each specified mRNA-LNP. The number of immunizations and dose of mRNA-LNP was based on previous studies and experience with mRNA-LNP vaccination (8, 10). Group 1 received 10 μg mRNA-LNP encoding for CCHFV glycoproteins (GcGn), group 2 were immunized with 10 μg mRNA-LNP encoding for the viral nucleoprotein (N), group 3 was given both 10 μg GcGn and 10 μg N mRNA-LNP, and group 4 were immunized with 10 μg control mRNA-LNP containing poly(C) RNA. The injection was divided over four points on the back of the mice and each injection created a small elevation of the skin, which disappeared within 5 min after immunization. No other noticeable clinical signs were observed due to immunization. Five weeks after the second immunization (at an age of 14 to 16 weeks), all animals were challenged with a lethal dose of CCHFV IbAr10200 and monitored for 14 days. The health status of the animals was checked daily and evaluated based on several parameters, including general condition, piloerection, as well as movement and posture. While the control animals all showed signs of severe disease and reached the humane endpoint 4 days postinfection, none of the vaccinated animals showed any clinical symptoms and all survived until the end of the study (Fig. 1c). At the time of euthanization, the control mice had lost an average of 13.4 % ± 2.0% of their body weight compared with the day of challenge. In contrast, animals in the GcGn, GcGn+N, and N vaccine groups had gained an average of 0.8% ± 1.7%, 0.8% ± 0.5%, and 4.2% ± 2.5% body weight, respectively, at the end of the study compared with the day of challenge. The slightly higher weight gain observed in the N-group was mostly due to two mice gaining 1.2 g and 1.9 g, respectively, compared with the day of challenge. This increase in weight deviated some from the rest of the surviving mice whose average weight gain was 0.3 g ± 0.3 g. Viral RNA in serum, liver, and spleen were measured using quantitative real-time PCR (qRT-PCR). All mice in the control group clearly had detectable CCHFV RNA in all tissues tested (Fig. 1d to f). In contrast, most animals in the vaccinated groups had undetectable levels of viral RNA in serum (Fig. 1d) and very low, but measurable levels, in spleen and liver (Fig. 1e and f). With an average cycle threshold (CT) value in serum of 26.85 for control animals compared with undetectable (CT ≥ 40.00) in the GcGn-, 37.98 in the N-, and 39.09 in the GcGn+N group (only three and two out of six mice in the N- and the GcGn+N groups, respectively, had detectable viral RNA in serum), there were significantly (*P* < 0.0001) lower CCHFV RNA levels in serum from all three immunized groups compared with the control group. Similarly, when analyzing spleen and liver, there were significantly lower levels of viral RNA in both the spleen (*P* > 0.0001) and liver (*P* > 0.0001) of CCHFV mRNA-LNP-vaccinated animals compared with the control group (Fig. 1e and f). These results show that immunized IFNAR^−/−^ mice survive and are able to control the infection, but that the vaccination does not lead to sterilizing immunity.

### Immunization with nucleoside-modified mRNA-LNP elicits a strong humoral immune response.

**FIG 2:**
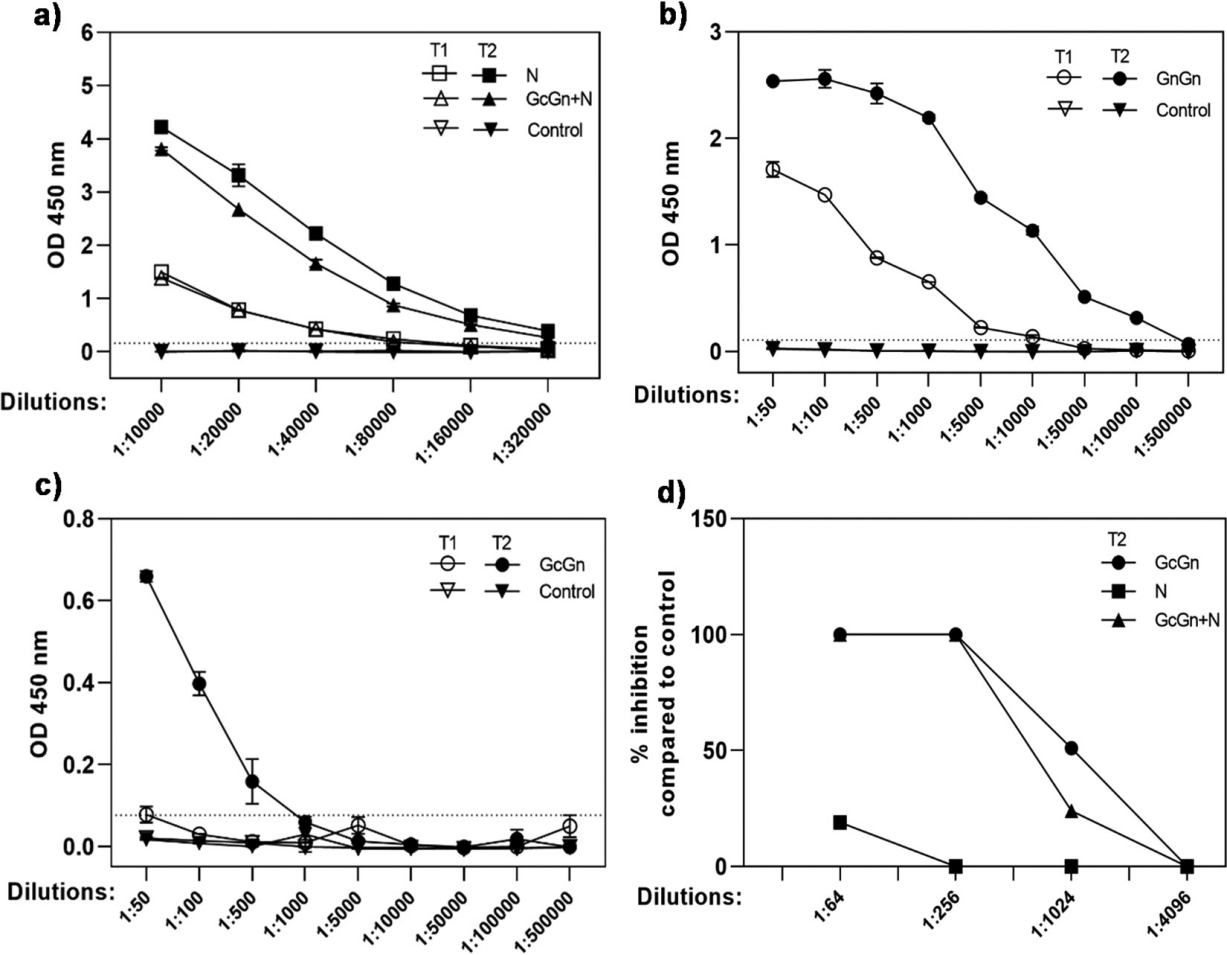
Antibody response in IFNAR^−/−^ mice due to mRNA-LNP vaccination. (a) Antibody titer in pooled, diluted serum from IFNAR^−/−^ mice immunized with N or GcGn+N mRNA-LNP after the first (T1) and second (T2) immunization measured with VectorBest ELISA. (b) Titers of antibodies against CCHFV Gc or (c) Gn in pooled, diluted serum from IFNAR^−/−^ mice immunized with GcGn mRNA-LNP measured with in-house ELISAs coated with CCHFV Gc or Gn protein, respectively. Data is shown as mean ± standard deviation of two technical replicates. Dotted lines indicate limit of detection. (d) Pooled, diluted serum from IFNAR^−/−^ mice for each vaccine group after two immunization (T2) were evaluated for neutralization capacity. Data is shown as percent inhibition compared with control animals for each dilution. T1 is represented by white symbols: N, GcGn+N, GcGn, control. T2 is represented by black symbols: N, GcGn+N, GcGn, and control. OD, optical density.

To examine if immunization induces antibody production against N and GcGn, antigen-specific antibody titers were measured using a commercial ELISA. For each immunization group and time point, serum was pooled and 2-fold serially diluted starting at 1:10,000. No antibodies were detected in preimmunization sera (T0) or sera from control animals at any time point tested (data not shown). In contrast, high levels were observed in the groups immunized with either N or GcGn+N mRNA-LNP after both one (T1) and two (T2) immunizations, with an increase in antibody levels after the second immunization (Fig. 2a). The antibody titer was 80,000 and 320,000 in both the N and the GcGn+N group after the first and second immunization, respectively.

Surprisingly, we could not detect antibodies in animals vaccinated with only GcGn mRNA-LNP using the commercial ELISA. To further investigate this observation, serum from mice in the GcGn group were pooled and analyzed using an in-house, indirect immunofluorescences assay (IFA) based on CCHFV-infected Vero cells. The staining (data not shown) showed that immunization with GcGn mRNA-LNPs induced antibodies. Due to this finding, and to distinguish between antibodies against the Gc- and Gn protein, we developed an in-house ELISA based on CCHFV recombinant Gc or Gn protein. As shown in Fig. 2b, just one immunization with GcGn mRNA-LNPs induced antibodies against CCHFV Gc with a titer of 5,000. After the second immunization, the titer increased to 100,000. In contrast, anti-Gn antibodies were only detected after the second immunization (Fig. 2c) at a 200 times lower titer (500) compared with anti-Gc antibodies.

Next, we assessed if the CCHFV-specific antibodies had neutralizing capacity by using a microneutralization assay. As shown in Fig. 2d, we found that mice immunized with GcGn or GcGn+N mRNA-LNP developed neutralizing antibodies, while N mRNA-LNP-vaccinated animals did not. Together these findings show that both GcGn and N mRNA-LNP immunization induces antibody production. While antibodies against Gc, and to some extent Gn, most likely contribute to protection against CCHFV infection by neutralization of the virus, protection induced by N mRNA-LNP immunization is mediated through another mechanism(s).

### CCHFV nucleoside-modified mRNA-LNP vaccination induces robust immune responses in immunocompetent mice.

Many innate immune receptors recognizes RNA and induce immune responses (28). Activation of pattern recognition receptors (PRR) due to RNA sensing leads to release of type 1 interferon, which can prompt inhibition of translation. Because the IFNAR^−/−^ mice lack type 1 interferon receptors, these mice do not respond to a potential IFN-α/β release and thus, translation is not blocked. Hence, to broaden the investigation regarding CCHFV mRNA-LNP-induced immune responses and to verify that the vaccine stimulates the same immune responses in immunocompetent mice as observed in IFNAR^−/−^ mice, 24 immunocompetent (129S2) mice were divided into four vaccination groups (*n* = 6) and immunized as shown in Fig. 1b. Antibody responses and neutralizing capacity were evaluated using the same methods as described above.

**FIG 3:**
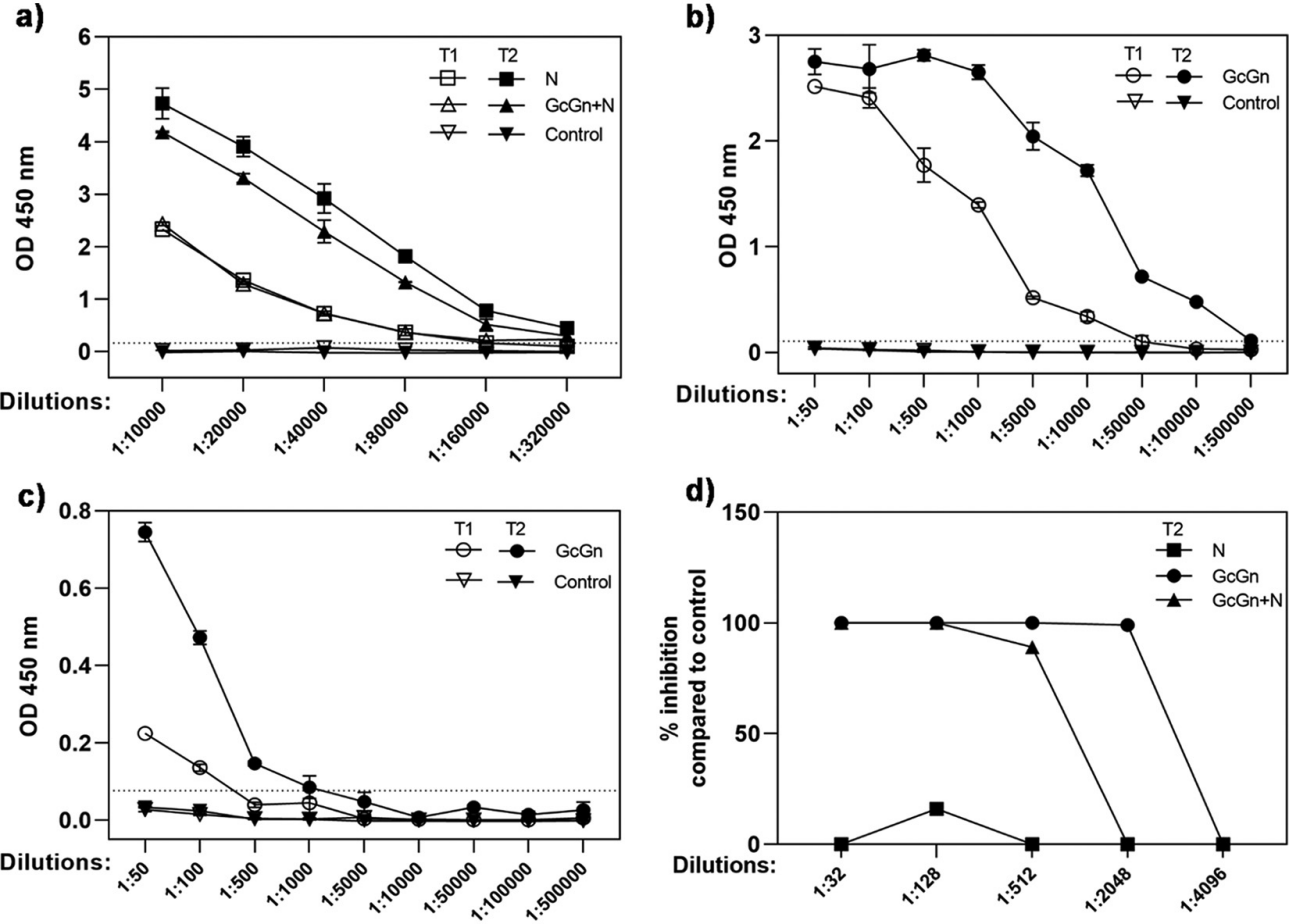
Antibody response in immunocompetent mice due to mRNA-LNP vaccination. (a) Antibody titer in pooled, diluted serum from immunocompetent mice immunized with N or GcGn+N mRNA-LNP after the first (T1) and second (T2) immunization measured with VectorBest ELISA. (b) Titers of antibodies against CCHFV Gc or (c) Gn in pooled, diluted serum from immunocompetent mice immunized with GcGn mRNA-LNP measured with in-house ELISAs coated with CCHFV Gc or Gn protein, respectively. Data is show as mean ± standard deviation of twp technical replicates. Dotted lines indicate limit of detection. (d) Pooled, diluted serum from immunocompetent mice for each vaccine group after two immunization (T2) were evaluated for neutralization capacity. Data is shown as percent inhibition compared with control animals for each dilution. T1 is represented by white symbols: N, GcGn+N, GcGn, control. T2 is represented by black symbols: N, GcGn+N, GcGn, and control. OD, optical density.

Similar to the knockout mice, immunocompetent mice immunized with either N mRNA-LNP alone or in combination with GcGn mRNA-LNP showed high levels of CCHFV-specific antibodies after one immunization (titer of 80,000), with an increase after the second immunization (titer of 320,000) (Fig. 3a). With the in-house ELISA, anti-Gc and anti-Gn antibodies were detected in serum from GcGn mRNA-LNP immunized mice (Fig. 3b and c). A high titer (10,000) of anti-Gc antibodies was measured after just one immunization with a 10-fold increase after the second immunization (Fig. 3b). In contrast to the IFNAR^−/−^ mice, low levels of anti-Gn antibodies were detected in the immunocompetent mice after one immunization with an increase after the boost (Fig. 3c). However, the anti-Gn titer was 200 times lower compared with anti-Gc antibodies.

Just as in the IFNAR^−/−^ mice, serum from GcGn and GcGn+N immunized immunocompetent mice showed neutralizing capacity (Fig. 3d). Serum from the N mRNA-LNP immunized group had no significant neutralizing effect.

**FIG 4:**
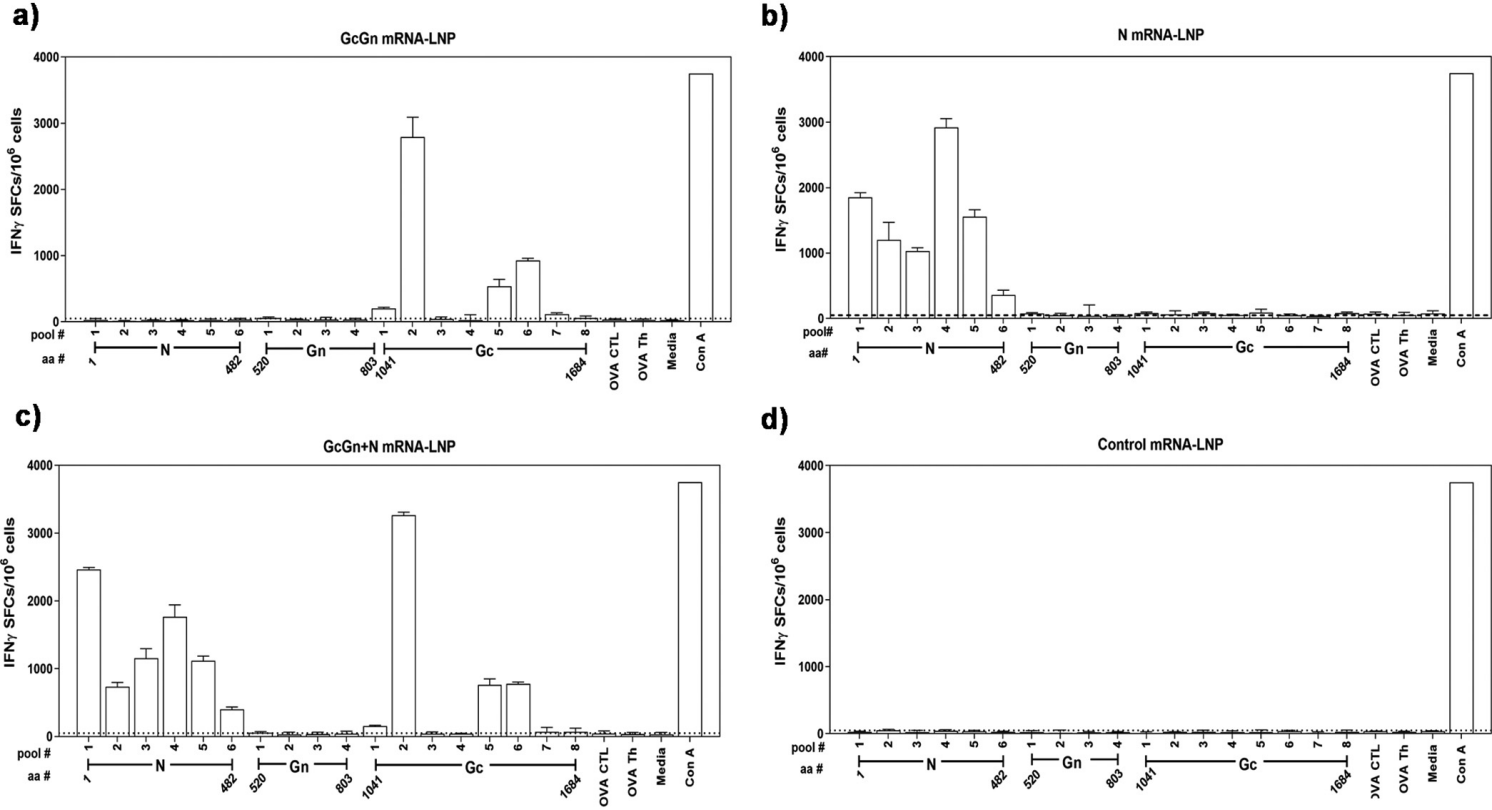
mRNA-LNP immunization induces CCHFV-specific cellular response. Fresh splenocytes from the immunocompetent mice after two immunizations were stimulated with pooled, overlapping peptides based on the CCHFV Gc, Gn, or N protein. ELISpot was used to determine the number of IFN-γ SFCs per 10^6^ splenocytes in (a) GcGn,(b) N, (c) GcGn+N, and (d) control vaccinated mice. Each peptide pool for N and Gc contained eight peptides, while Gn-peptide pools contained seven peptides. Each pool was performed in triplicate and data is shown as geometric mean ± geometric standard deviation. As control antigens OVA-CTL, OVA-Th, ConA, and medium alone were used and cut-off was set at 50 SFCs/10^6^ splenocytes. Amino acid position (aa#) is based on CCHFV nucleoprotein, NCBI accession number NP_950237 and CCHFV glycoprotein precursor, NCBI accession number NP_950235.

To further characterize the different vaccine candidates, we analyzed T-cell responses against CCHFV using IFN-γ ELISpot. Five weeks after the last immunization, fresh splenocytes from the immunocompetent mice in each vaccination group were pooled and incubated with peptides based on CCHFV IbAr10200 N, Gc, or Gn proteins. All groups showed activation of CCHFV-specific IFN-γ producing T-cells to peptide pools spanning the immunogens used (Fig. 4a to c), while control immunization did not induce cellular response (Fig. 4d). Immunization with GcGn mRNA-LNP generated a strong T-cell response to the Gc peptides (ranging from 118 to 2,802 SFCs/10^6^ splenocytes) and specifically to peptide pool #2 (amino acids 1121 to 1210, NP_950235) (Fig. 4a). Interestingly, no significant response to any of the Gn peptides was observed. Splenocytes from N mRNA-LNP-immunized mice reacted to all pools of N peptides and produced IFN-γ ranging from 363 to 2,922 SFCs/10^6^ splenocytes (Fig. 4b). In the GcGn+N-immunized group, T-cell responses to both N and Gc peptides was observed, but again, no T-cell activation due to incubation with Gn peptides (Fig. 4c). Control animals showed no response to either N, Gc, or Gn derived peptides (Fig. 4d). These results show clear generation of CCHFV specific cellular immune response by immunization with either N, GcGn, or N+GcGn mRNA-LNP, which could contribute to protection against infection.

### High throughput quantitative proteomics identified distinct proteomic vaccine signature linked to metabolism.

High throughput technologies have contributed to identification of early immune responses to vaccination and their role in protection against infection (29–32). Therefore, to identify and better understand more large-scale potential differences between the two vaccine candidates and, in addition, compare with control after CCHFV infection, we conducted a quantitative proteomic study. Liver samples from GcGn- or N mRNA-LNP-immunized, infected animals, as well as the control mice were included.

**FIG 5:**
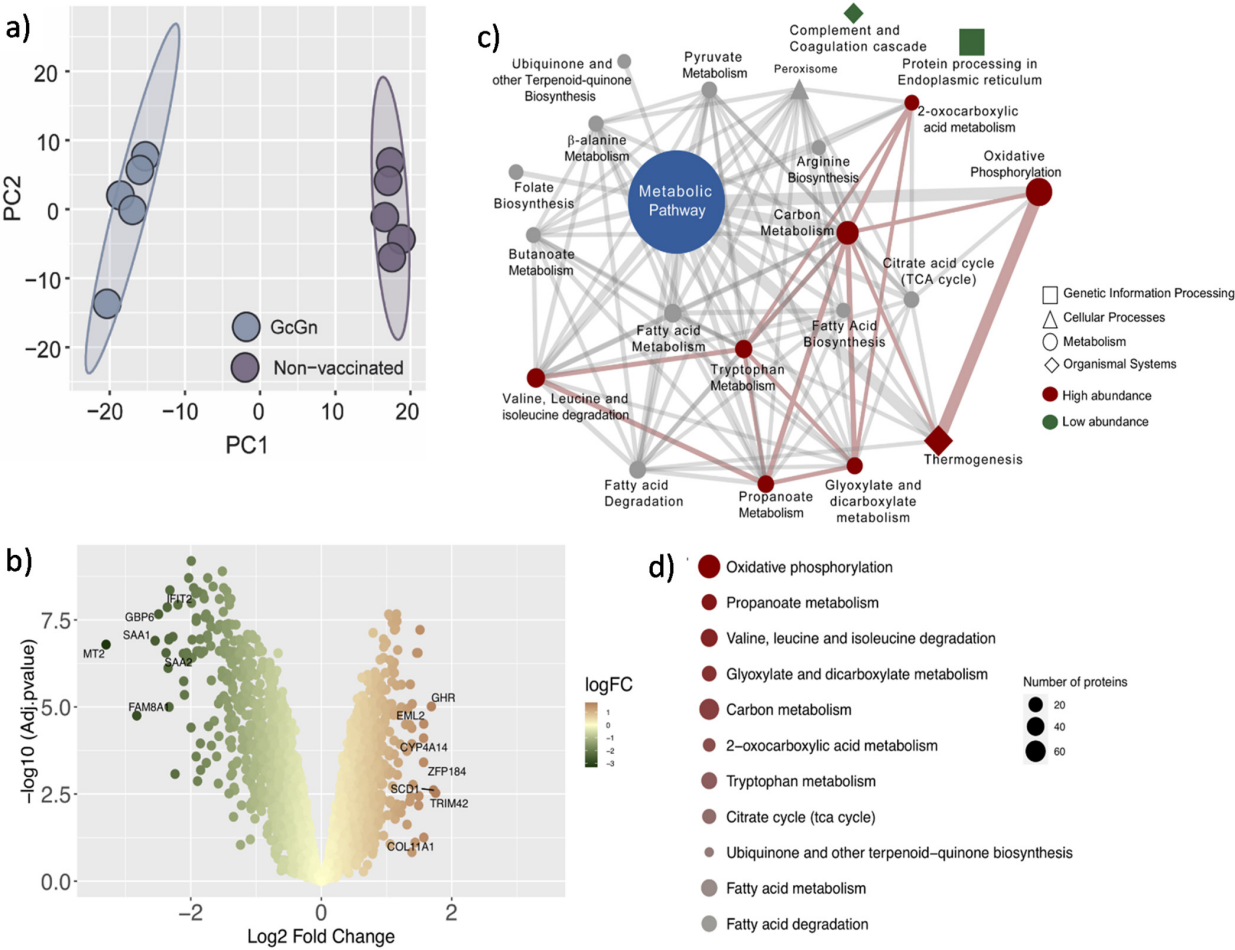
Proteomic analysis of GcGn-vaccinated versus control mice. (a) PCA trajectory score plots labeled for GcGn-vaccinated and control mice. (b) Volcano plots of proteins with differential abundance between GcGn-vaccinated and control mice. Upregulated proteins are represented in red, while proteins downregulated are represented in green. FDR < 0.05. (c) Cytoscape network of KEGG pathways enriched in proteins differing between GcGn-vaccinated and control mice. Nonsignificant pathways are gray. Significant upregulated pathways are represented in red and downregulated in green. Node size is proportional to the number of proteins associated with a pathway. Edge width is proportional to the number of proteins shared between two pathways. (d) Bubble plot representing significant pathways from the KEGG pathway map “Metabolism” enriched in proteins with significant higher abundance in GcGn vaccinated mice compared with control. Pathways are ordered by adjusted *P value* and size is proportional to the number of proteins.

Principal component analysis (PCA) showed a clear separation between GcGn-vaccinated, infected mice and control-vaccinated, infected mice (Fig. 5a), and differential protein abundance analysis identified 1,288 proteins with higher abundances and 1,079 proteins with lower abundance between the two groups (Fig. 5b, Data Set S1). Protein set enrichment analysis (PSEA) using the Kyoto Encyclopedia of Genes and Genomes (KEGG) linked most of the high abundance proteins in the GcGn-vaccinated mice to eight pathways (adjusted *P* < 0.05), while the low abundance proteins belonged to two pathways (adjusted *P* < 0.05). Most of the effected proteins where connected to, “metabolic pathways,” which even though not significantly changed, indicate a shift in metabolism due to GcGn mRNA-LNP vaccination (Fig. 5c). Thus, PSEA was performed only on pathways from the KEGG map pathway “Metabolism” in order to identify additional mechanism shifts in GcGn-vaccinated mice (Fig. 5d). Top pathways differing between GcGn and control mice were oxidative phosphorylation, propanoate metabolism, and valine, leucine, and isoleucine degradation.

**FIG 6:**
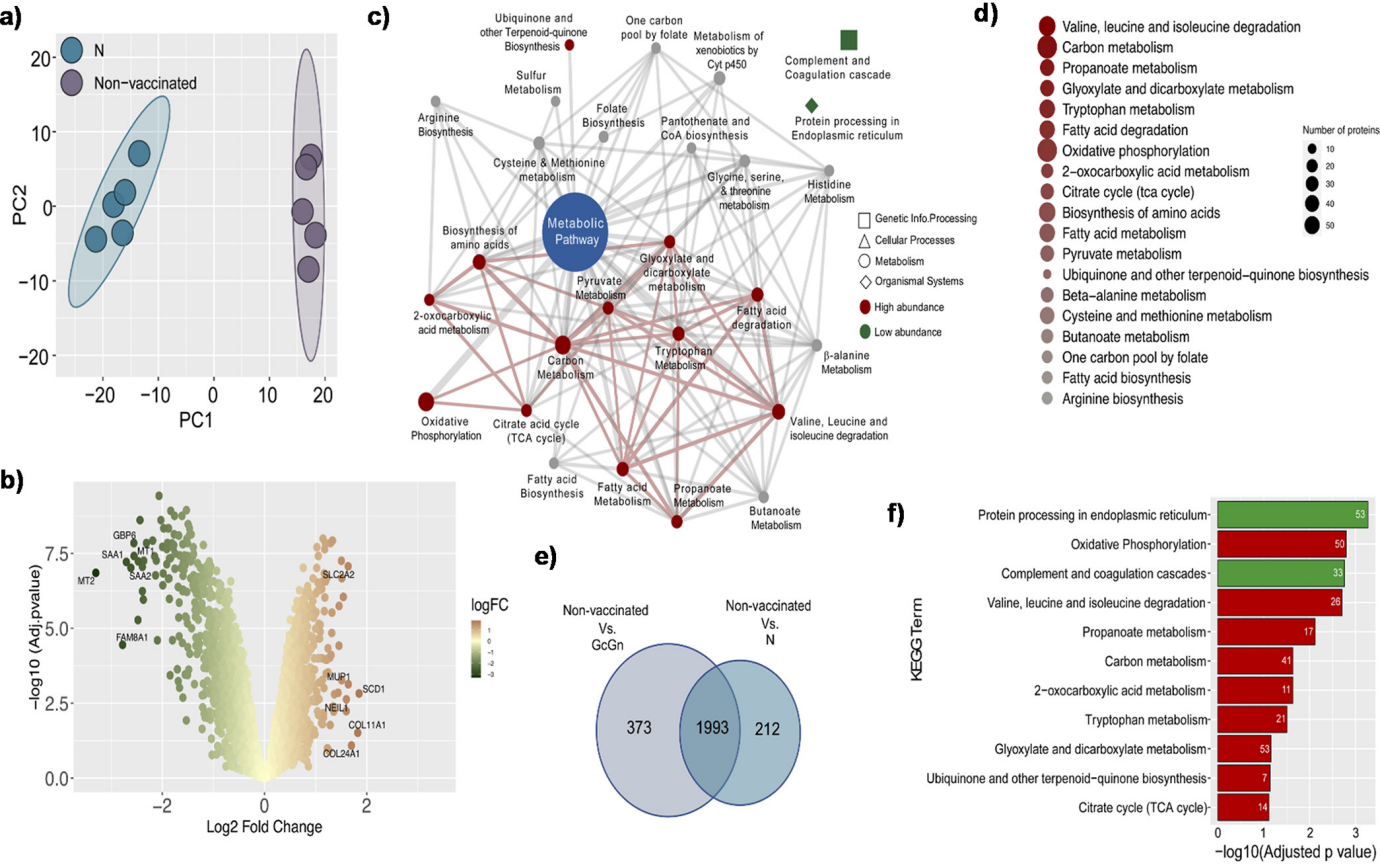
Proteomics analysis of N-vaccinated versus control mice. (a) PCA trajectory score plots labeled for N-vaccinated and control mice. (b) Volcano plots of proteins with differential abundance between N-vaccinated and control mice. (c) Cytoscape network of KEGG pathways enriched in proteins differing between control and GcGn. (d) Bubble plot representing KEGG pathways from the “Metabolism” pathway map enriched in the protein with higher intensity in N-vaccinated mice compared with control. (e) Venn diagram of protein with differential abundance between control, GcGn-, and N-vaccinated mice. (f) Barplot of the top 10 biological KEGG pathways enriched in proteins differing between control and, respectively, GcGn- and N-vaccinated groups of mice. Pathways were ordered by adjusted *P value*. Upregulated pathways are represented in red and downregulated pathways in green. Number of proteins associated with each pathway is indicated on the bar.

Similar analysis was performed comparing N-vaccinated, infected mice to control-vaccinated, infected mice, and PCA plots identified separate clustering of N-vaccinated mice verses control (Fig. 6a). When comparing N-vaccinated mice to control mice, differential protein abundance analysis identified 1,218 proteins with high abundance and 988 proteins with low abundance (Fig. 6b, Data Set S1). With PSEA, 12 pathways (adjusted *P* < 0.05) with high abundance proteins and two pathways (adjusted *P* < 0.05) with low abundance proteins were identified in N-vaccinated mice compared with controls. As in previous comparison with GcGn-vaccinated mice, most proteins were associated with “metabolic pathways” (Fig. 6c). PSEA on the KEGG “metabolism” map pathway revealed that the most significant mechanisms differing between N-vaccinated and control mice were changes in the amino acid metabolism (Fig. 6d). Specifically, valine, leucine, and isoleucine degradation, as well as carbon and propanoate metabolism.

When comparing the two different vaccine candidates, we identified 1,993 proteins to be differently regulated in vaccinated mice (both GcGn and N) compared with control mice (Fig. 6e). These 1,993 proteins were submitted to PSEA and the top 10 pathways were extracted (Fig. 6f, Data Set S2). The top pathways were oxidative phosphorylation, valine, leucine, and isoleucine degradation, propanoate metabolism, and carbon metabolism where the proteins were highly abundant in vaccinated mice, confirming the pairwise analysis. The pathways where most of the proteins were low abundance belongs to the protein processing in endoplasmic reticulum and complement and coagulation cascades. No proteins differed between GcGn- and N-vaccinated mice indicating minimal changes in the protein abundance in the two vaccination strategies.

## DISCUSSION

In this study, using the nucleoside-modified mRNA-LNP vaccine platform, which have shown powerful and very promising results in the development of a vaccine against COVID-19 (17–25), we demonstrate 100% protection of IFNAR^−/−^ mice against lethal CCHFV infection. The mice received immunization with mRNA-LNP encoding for either CCHFV glycoproteins or the nucleoprotein, and protection against death and disease was seen with both. In addition, we show induction of robust humoral and cellular immune responses in both IFNAR^−/−^ and immunocompetent mice due to immunization. Also, to our knowledge, this is the first time large-scale analysis of CCHFV vaccine-induced effects, in combination with infection, on the protein profile have been conducted using proteomics. The results indicate a metabolic sparing in the liver of vaccinated mice with no significant difference between animals immunized with N or GcGn mRNA-LNP.

In a previous study, Aligholipour Farzani et al. assessed immunogenicity and protection against CCHFV of a naked mRNA vaccine encoding the viral nucleoprotein after one or two immunizations. They report 100% protection with a booster strategy, while with one immunization the mice developed severe symptoms and only 50% survived. Despite survival, their data show weight loss postinfection in both vaccine groups in the range similar to that observed in the control group. In our study, the control group showed signs of disease and were euthanized 4 days postinfection with an average weight loss of 13.4% ± 2.0%. While developing a vaccine that prevents death is a success, our aim is to develop a vaccine that also protects from severe disease. While we observed an increase in antibody titer with a second immunization, Aligholipour Farzani et al. did not. This dissimilarity might be due to differences in the vaccine platforms used. We used nucleoside-modified mRNA encapsulated in lipid-containing nanoparticles, while Aligholipour Farzani et al. used naked mRNA, which are more quickly degraded by extracellular RNases and thus might lead to a weaker immune response.

Even though immunization in the current study, independent of CCHFV vaccine candidate, protected from disease, it did not induce sterilizing immunity as viral RNA was detected in some of the immunized mice (Fig. 1d to f). However, it is not determined if the RNA represent infectious virus and the level was at least 3-fold lower than control mice (Fig. 1f). Interestingly, we found that mice immunized with GcGn mRNA-LNP had no viral RNA in serum, indicating efficient levels of neutralizing antibodies and/or other immune factors to clear the virus from the blood in these mice. One caveat with our study is that samples for PCR from the vaccinated mice was not collected at the same time as for the control group. The purpose of the study was to investigate protection from disease and thus, the vaccinated mice had to be observed for a longer time to rule out the possibility that vaccination prolonged the incubation period of the disease. It is possible that viral levels were higher in the vaccinated mice early in infection. Unfortunately, the regulations regarding what we can do with infected mice in our BSL-4 laboratory is limited and, therefore, we were not able to collect blood from living mice inside of the BSL-4.

Independent of vaccine candidate, all immunized mice, (both IFNAR^−/−^ and immunocompetent), showed strong antibody production after only one immunization (Fig. 2a to c and 3a to c), with an increase after the boost. By conducting ELISAs detecting antibodies against Gc and Gn separately, we showed that GcGn vaccination induced much higher titer of anti-Gc antibodies compared with anti-Gn antibodies (Fig. 2b to c and 3b to c). This is consistent with previous studies showing CCHFV Gc to be more immunogenic compared with Gn (33). However, even though the Gn protein does not induce a strong immune response, it is most likely necessary to include in a vaccine against CCHFV. Several proteins derived from the CCHFV M polyprotein, including the Gc protein, depend on the Gn protein for correct localization, folding, and transport (34).

Furthermore, we have studied T-cell response due to the mRNA-LNP immunization in detail and showed that both N and GcGn mRNA-LNP induced priming of T-cells against CCHFV (Fig. 4a to c). Because interaction with all pools of N-based peptides lead to IFNɣ production, the result suggests that a large part of the N protein can act as an antigen. In contrast, for the glycoprotein, four out of eight Gc-peptide pools induced IFN-γ production, with pool #2 (amino acids 1121 to 1210 of NCBI accession number NP_950235), inducing the highest number of IFN-γ SFCs/10^6^ cells. Peptides based on the Gn protein did not activate T-cells, further demonstrating Gn to be less immunogenic than Gc. Similar results were observed in a previous study investigating long-lived T-cell responses due to CCHFV infection in humans (35). T-cells from CCHF survivors were activated by interaction with CCHFV peptides, with the reactive peptides predominantly located in the nucleoprotein. Only one survivor reacted to epitopes from the Gc protein and no reaction to the Gn protein was observed. Thus, our immunization candidates induce a similar T-cell response as prompted by natural CCHFV infection.

There is currently no data available on the effect of immunization on the proteomic profile in mice recovering from CCHFV infection. Here, when comparing the proteomic profile of mice immunized with either GcGn or N mRNA-LNP, no significant difference was observed. On the other hand, when comparing vaccinated and control mice after CCHFV infection, a distinct metabolic shift toward a loss in oxidative phosphorylation; valine, leucine, and isoleucine degradation; and propanoate and carbon metabolism was identified in control mice. These pathways are often impaired in viral infection (36–38). Interestingly, proteins related to the complement and coagulation cascade were upregulated in control mice compared with vaccinated animals.

The results from the current study showing survival of mice immunized with only N mRNA-LNP strongly indicates that neutralizing antibodies are not necessary for protection against CCHF. This supports previous results from our group presenting no correlation between protection and neutralizing antibody titer (27). Immunization with a DNA plasmid encoding the CCHFV glyco- and nucleoprotein fully protected IFNAR^−/−^ mice against CCHF, while immunization with transcriptionally competent virus-like particles (tc-VLPs) only led to 40% survival despite induction of higher titer of neutralizing antibodies compared with the DNA vaccine. Similar, Kortekaas et al. showed production of neutralizing antibodies in STAT1 knockout mice with a CCHFV glycoprotein based vaccine, but no protection against infection (33). Recently, we published a study showing protection against CCHF in Cynomolgus macaques immunized with a DNA vaccine based on the CCHFV glyco- and nucleoprotein (39). Similar to the current study, vaccination led to induction of both CCHFV specific antibodies and T-cells. However, the antibodies were mainly against the N protein and, thus, lacked neutralizing capacity.

Even though no neutralizing antibodies, but a strong T-cell response, was detected in immunocompetent N mRNA-LNP immunized mice, we cannot conclude that only a cellular immune response results in an efficient protection against CCHFV infection because T-cell responses were not evaluated in the IFNAR^−/−^ mice. To determine if N mRNA-LNP-mediated cellular immunity is enough to prevent CCHFV infection, T- and B-cell depletion or serum transfer studies have to be conducted in the IFNAR^−/−^ model.

In conclusion, we show that immunization with nucleoside-modified mRNA-LNP encoding for CCHFV nucleoprotein or glycoproteins both effectively protect IFNAR^−/−^ mice from lethal infection of CCHFV, thus the question regarding the best vaccine candidate remains. Even though vaccination with GcGn alone protected mice from disease, and induced both CCHFV neutralizing antibodies and primed T-cells, a vaccine including the nucleoprotein might still be preferred, due to the immunogenicity of the N protein, but also because of its more conserved sequence between different CCHFV strains compared with the glycoprotein (1). Thus, including the nucleoprotein in the vaccine could increase the chance of protection against infection with different CCHFV strains. Based on the current study and additional successful studies against other infectious diseases, not least the development of a vaccine against SARS-CoV-2, using the mRNA-LNP platform, we believe we have necessary evidence to bring this vaccine platform to the next step in development of a vaccine against CCHFV infection.

## MATERIALS AND METHODS

### Ethical statement.

In the current study, we used female A129 IFNAR^−/−^ mice (Marshall Bioresources, Hull, UK) and 129S2 immunocompetent mice (Envigo, Venray, the Netherlands) at an age of 6 to 8 weeks at the time of the first immunization. The animals were housed according to Karolinska Institute ethical rules and observed daily. Stockholm Ethical Committee for animal research approved the research.

### Production of mRNA lipid-nanoparticles.

mRNAs were produced as previously described (40) using T7 RNA polymerase on linearized plasmids (pUC-TEV-CCHFV GcGn-A101, pUC-TEV-CCHFV N-A101) encoding codon-optimized CCHFV glycoproteins and the nucleoprotein protein from the IbAr10200 CCHFV strain. mRNAs were transcribed to contain 101 nucleotide-long poly(A) tails. Instead of UTP, 1-methylpseudouridine-5′-triphosphate (TriLink) was used to generate modified nucleoside-containing mRNAs. mRNAs were capped using the m7G capping kit with 2’-*O*-methyltransferase to obtain cap1 and were purified by cellulose purification, as described earlier (41). mRNAs were analyzed by agarose gel electrophoresis and stored frozen at −20°C.

Cellulose-purified mRNAs and poly(C) RNA (Sigma-Aldrich, St. Louis, MO) were encapsulated in LNP using a self-assembly process in which an aqueous solution of mRNA at pH 4.0 is rapidly mixed with a solution of lipids dissolved in ethanol (42). LNP used in this study were similar in composition to those described previously (42, 43), which contain an ionizable cationic lipid (proprietary to Acuitas). The proprietary lipid and LNP composition are described in U.S. patent US10221127. They had a diameter of ∼80 nm as measured by dynamic light scattering using a Zetasizer Nano ZS (Malvern Instruments Ltd, Malvern, UK) instrument.

### mRNA transfection and protein analysis.

The *in vitro* synthesized and purified mRNA was transfected into HEK293T cells using TransIT-mRNA (MirusBio, Madison, WI) as described previously (7). Briefly, 6 × 10^4^ cells were transiently transfected with 0.3 μg of RNA in combination with 0.34 μL TransIT-mRNA reagent and 0.22 μL boost reagent in 17 μL of serum-free medium. At 16-h posttransfection, cells were lysed in 100 μL of 1x cell lysis buffer (20 mM Tris-HCl (pH 7.5), 150 mM NaCl, 1% Triton) with complete protease inhibitor cocktail. Whole cell lysates from the transfected cells were separated on a 4% to 12% Bis-Tris polyacrylamide gel in MOPS electrophoresis buffer (Life Technologies, Carlsbad, CA) and blotted to nitrocellulose membranes using the iBlot2 Dry Blotting System (Life Technologies). The membranes were washed with 1× Tris-buffered saline (TBS) and blocked in TBS buffer containing 5% nonfat, dry milk and 0.5% Tween 20. Primary polyclonal antibodies against CCHFV Gn, Gc, and N proteins were diluted in 1× Tris-buffered saline with 0.1% Tween 20 (TBST) and membranes incubated at room temperature (RT) overnight with agitation. Recombinant anti-vinculin antibody or anti-alpha tubulin antibody (Abcam, Cambridge, UK) were used as loading controls. After the final washing, expression of CCHFV Gn (37 kDa), Gc (75 kDa), and N (55 kDa) proteins were visualized using LI-COR C-DiGit Blot Scanner.

### Virus.

The titer of CCHFV strain Nigerian IbAr10200 (number of passages not known) (44) was determined on Vero cells. The virus was serially diluted 10-fold and then added to Vero cells in a 96-well plate. Twenty-four-h postinfection, cells were fixed with ice-cold acetone-methanol (1:1) and incubated for at least 30 min. Thereafter, the cells were stained with a primary antibody (1:150) made in rabbits against the CCHFV nucleoprotein for 30 min at 37°C followed by staining with secondary antibodies anti-rabbit Alexa Fluor 488 (Thermo Fisher Scientific, Waltham, MA) and DAPI (Roche, Mannheim, Germany) (1:1,000). The number of fluorescent foci in each well was counted, and the titer determined.

### Immunizations and challenge.

The IFNAR^−/−^ mice were divided into four groups (*n* = 6) and immunization was performed twice with a 3-week interval. Mice receiving only N, GcGn, or the control (PolyC) mRNA-LNP were injected intradermally at four different points on their back with a total of 10 μg mRNA-LNP in 120 μL calcium and magnesium-free phosphate-buffered saline (PBS), while mice immunized with both N and GcGn received a total of 20 μg mRNA-LNP in 120 μL PBS. Blood samples were collected 2 days prior to the first immunization, right before the second immunization and before viral challenge. In addition, at the time of euthanasia, blood, liver, and spleen were collected from each mouse. The blood samples were collected in serum-separation tubes, centrifuged, and serum, spleen, and liver were frozen until use. Five weeks post the last immunization, all IFNAR^−/−^ mice were challenged with 400 focus forming units (ffu) of the CCHFV strain Nigerian IbAr10200 (titer of 1 × 10^4^ ffu/ml) in 100 μL by intraperitoneal injection. After infection, the animals were monitored up to 14 days with respect to clinical signs of disease, their overall well-being, and survival. Furthermore, 24 immunocompetent mice (129S2) were immunized as the IFNAR^−/−^ mice described above. Blood samples were collected before and prior to both immunizations. Five weeks after the last immunization, animals were euthanized and blood and spleen collected.

### Viral RNA detection.

To inactivate virus in serum, TRIzol was added to the samples (1:3). For liver and spleen, PBS was added to each sample and pestles were used to crush the organs. Thereafter, the samples were centrifuged (5 min at 7,000 rpm) and 100 to 200 μL of each liver or spleen sample was added to TRIzol (1:3). RNA was extracted using the Direct-zol RNA Miniprep kit (Zymo Research, Irvine, CA) according to the manufacturer’s instructions. Viral RNA was measured by quantitative real-time PCR (qRT-PCR) using TaqMan Fast Virus 1-Step Master Mix (Thermo Fisher Scientific) with primers and probe specific for the CCHFV L gene:

Forward, 5′- GCCAACTGTGACKGTKTTCTAYATGCT-3′,

Reverse 1, 5′- CGGAAAGCCTATAAAACCTACCTTC-3′,

Reverse 2, 5′-CGGAAAGCCTATAAAACCTGCCYTC-3′ and

Reverse 3, 5′-CGGAAAGCCTAAAAAATCTGCCTTC-3′, and

Probe, FAM-CTGACAAGYTCAGCAAC–MGB.

For liver and spleen samples, mouse ACTB mix (Thermo Fisher Scientific) was used as endogenous control. The cycling reactions was performed using a capillary Roche LightCycler 2.0 system.

### VectorBest and in-house enzyme-linked immunosorbent assay.

Antibodies against CCHFV were measured using either a commercial ELISA (VectorBest, Novosibirsk, Russia) or an in-house ELISA based on the CCHFV Gc or Gn proteins. For the VectorBest ELISA, the manufacturer’s protocol was followed with minor modifications. Dilution buffer from the ELISA kit and a murine monoclonal CCHFV antibody against the nucleoprotein were used as negative and positive control, respectively, and included on every plate. As a secondary antibody, horseradish peroxidase conjugated polyclonal rabbit anti-mouse immunoglobulin (Agilent, Santa Clara, CA) was used at a dilution of 1:8,000. For the in-house ELISA, maxiSorp 96-well plates were coated with either recombinant CCHFV Gc or Gn protein (The NativeAntigen Company, Oxford, UK). The proteins were diluted to 1 μg/mL in sodium carbonate buffer and 100 μL was added to each well before incubation overnight at RT. Plates were then blocked (PBS with 1% BSA) for 2 h at RT and thereafter washed (PBS with 0.05% Tween 20). Serum samples were diluted in PBS with 1% BSA and 0.05% Tween 20, 100 μL added to each well and plates incubated at RT for 1 h. Thereafter, plates were washed and the same secondary antibody as used for the VectorBest ELISA was added at the same dilution. After washing, 100 μL of 3,3′,5,5′-tetramethylbenzidine (TMB) substrate was added to each well and the reaction was stopped after 4 min with 100-μL TMB stop reagent. Both the VectorBest and in-house ELISA was measured at 450 nm using a Multiskan FC Microplate Photometer. Cut-off for positive samples were based on serum before immunization (time point 0) and set to the average optical density value of all samples ± 2 standard deviations measured for each ELISA.

### Immunofluorescent staining.

Serum from before or after two immunizations (T2) of the GcGn mRNA-LNP immunized IFNAR^−/−^ mice was pooled and diluted 1:20. Thereafter, the diluted serum was added to CCHFV IbAr10200-infected Vero cells on glass slides and incubated in 37°C for 1 h. Serum was then decanted and slides washed in PBS three times for 5 min each before staining with Alexa 488 Goat anti-mouse antibody (1:1,000) (Thermo Fischer Scientific) and DAPI (1:1,000) (Roche). Slides were incubated for 30 min in 37°C before being analyzed by florescent microscopy (Nikon Eclipse TE300). Pictures were taken at 40 times magnification with an ORCA-spark Digital CMOS camera.

### Neutralization assay.

The titer of CCHFV-neutralizing antibodies in the serum of vaccinated IFNAR^−/−^ or immunocompetent mice was determined by microneutralization assay. Serum from all animals in each group were pooled and heat inactivated for 30 min at 56°C. Serial 2-fold dilutions of serum were mixed with approximately 120 CCHFV IbAr10200 particles and incubated at 37°C for 1 h. Thereafter, 100 μL of the serum-virus mix was added in duplicate to Vero cells on a 96-well plate (1.5 × 10^4^ cells/well). After 1-h incubation at 37°C, the inoculum was removed, cells were washed three times with Dulbecco’s Modified Eagle Medium (DMEM) supplemented with 2% FBS before 100 μL DMEM supplemented with 2% FBS was added and the cells were incubated 48 h at 37°C, 5% CO_2_. Cells were fixed with ice-cold acetone-methanol (1:1) in 4°C overnight and stained for CCHFV nucleoprotein for enumeration of fluorescent foci. Total numbers of infected cells in each well were counted and the results were expressed as percent neutralization compared with infection in wells with serum from the control group for each dilution.

### Peptides.

A total of 129, 20-amino-acid-long peptides with 10-amino-acid-overlap covering the CCHFV IbAr10200 N, Gc, and Gn sequences used for the different vaccine candidates (28 based on Gn, 64 based on Gc, and 48 based on N) were purchased from Sigma-Aldrich, Sweden. Peptides representing each viral protein was pooled, with N- and Gc- pools containing eight peptides, while Gn-peptide pools contained seven peptides.

### IFN-ɣ ELISpot.

Splenocytes from immunized immunocompetent mice were pooled based on each immunization group and tested for the presence of CCHFV specific T-cells by evaluating production of IFN-ɣ using Enzyme-linked ImmunoSpot (ELISpot). The assay is described previously (45), but briefly, ELISpot plates (Mabtech AB, Stockholm, Sweden) were pre-wet with 50 μL 70% ethanol for 1 min before washing with PBS and coated with 10 μg/mL of anti-mouse IFN-ɣ (Mabtech AB) in 100 μL per well. After overnight incubation at 4°C, plates were washed with PBS and blocked with complete medium (RPMI 1640 supplemented with 10% FBS, 1% GlutaMAX-I, 1% HEPES, 1% nonessential amino acids, 1% Penicillin-Streptomycin, and 0.0001% 2-mercaptoethanol) for 2 h at 37°C. Thereafter, 100 μL of each pool of specific peptides (total concentration 80 μg/mL) was added to designated wells and mixed with 200,000 fresh splenocytes in 100 μL complete RPMI 1640 medium. Plates were incubated for 48 h at 37°C, 5% CO_2_, washed, and 100 μL biotinylated anti-IFN-γ antibody (Mabtech AB) at a concentration of 2 μg/mL was added to each well. After incubation for 2 h at 37°C, the plates were washed thoroughly with PBS and incubated 1 h in RT with 100 μL per well of streptavidin-ALP (1:1,000). Thereafter, plates were washed again and 100 μL of AP conjugate substrate was added to each well and incubated for 10 min. Plates were rinsed with tap water and left to dry before measuring IFN-ɣ producing cells using the AID iSpot (EliSpot/FluoroSpot) reader system (Autoimmun Diagnostica GmbH, Straßberg, Germany). Concanavalin-A (1 μg/mL) was used as a positive control, ovalbumin-CTL peptide (1 μg/mL) and ovalbumin-Th peptide (1 μg/ml) as peptide controls, while RPMI 1640 medium was used as negative control. Spot counts were calculated as a mean number of spot forming cells (SFCs)/10^6^ cells and a mean number of IFN-γ–producing cells >50 SFC/10^6^ cells was considered as a positive result.

### Proteomics.

Parts of the liver from all infected IFNAR^−/−^ mice were crushed in 150 μL lysis buffer (10 mM Tris, 150 mM NaCl, 10% SDS, and protease inhibitor) using pestles. Then, samples were incubated at 4°C for 30 min and centrifuged at 13,000 rpm at 4°C for 20 min. Supernatant was collected, NuPAGE™ LDS sample buffer with 2-mercaptoethanol added and the samples boiled at 99°C for 10 min. Protein concentration for each sample was determined using Pierce™ 660 nm protein assay (Thermo Fisher Scientific). Tandem mass tag (TMTpro™, Thermo Fisher Scientific) based quantitative deep proteomic analysis, using nano-flow liquid chromatography hyphened to tandem mass spectrometry (LC-MS/MS), was performed in Proteomics Biomedicum, Karolinska Institute as described previously (46).

### Bioinformatics analysis.

Protein raw data abundance was first filtered for empty rows with an in-house script resulting in 5,168 proteins and normalized with R package NormalizerDE. Normalization with quantile was selected based on NormalizerDE report. In order to identify proteins changing with vaccination, differential abundance analysis was performed using R package LIMMA between control, GcGn, and N groups. Pairwise comparisons were extracted and Benjamini-Hochberg (BH) adjustment were applied on *P values*. Proteins with adjusted *P values* < 0.05 were selected. Results of the differential protein abundance were represented using Venn diagram from interactivenn (http://www.interactivenn.net/). The volcano plots to show the differently abundant proteins were performed using R package ggplot2. Up- and down-regulated proteins were submitted separately to gene set enrichment analysis (GSEA) using python module gseapy v0.9.17. Two manually curated KEGG libraries from the KEGG mouse 2019 (https://www.kegg.jp) were used, first where pathway maps “Human Diseases” and “Drug Development” were removed and then the pathway map “Metabolism” was conserved. Results were represented respectively as a network using Cytoscape ver 3.6.1 (https://cytoscape.org/) and bubble plot using ggplot2.

### Data availability.

The mass spectrometry proteomics data have been deposited in the ProteomeXchange Consortium via the PRIDE (47) partner repository with the data set identifier PXD023300 (https://www.ebi.ac.uk/pride/archive/projects/PXD023300).
